# Protein-A immunoadsorption combined with immunosuppressive treatment in refractory primary Sjögren’s syndrome coexisting with NMOSD: a case report and literature review

**DOI:** 10.3389/fimmu.2024.1429405

**Published:** 2024-07-11

**Authors:** Wei Fan, Xuyan Chen, Pingping Xiao, Bo Wei, Yi Zhang, Jinmei Huang, Shufan Wu, Liangjing Lu

**Affiliations:** ^1^ Department of Rheumatology and Immunology, The Second Affiliated Hospital of Xiamen Medical College, Xiamen, China; ^2^ Department of Rheumatology, Ren ji Hosptial, Shanghai Jiaotong University School of Medicine, Shanghai, China; ^3^ Department of Rheumatology, Zhongshan Hospital of Xiamen University, School of Medicine, Xiamen University, Xiamen, China

**Keywords:** immunoadsorption, treatment, Sjögren’s syndrome, NMOSD, case report

## Abstract

The treatment of primary Sjögren’s syndrome (pSS) coexisting with neuromyelitis optica spectrum disorder (NMOSD) using protein-A immunoadsorption combined with immunosuppressive therapy has rarely been reported. Herein, we present the case of a 35-year-old female diagnosed with pSS concomitant with NMOSD (pSS-NMOSD) who demonstrated a positive response to protein-A immunoadsorption after failing to respond to therapy comprising high-dose intravenous methylprednisolone (IVMP) and intravenous immunoglobulin (IVIG). Within one week of receiving three sessions of immunoadsorption combined with immunosuppressive treatment, the patient’s clinical symptoms (blurred vision, paraparesis, and dysfunctional proprioception) significantly improved. Additionally, a rapid decrease in the circulating levels of Aquaporin-4 immunoglobulin G antibodies (AQP4-IgG), immunoglobulin (Ig) A, IgG, IgM, erythrocyte sedimentation rate (ESR), and rheumatoid factor (RF) were observed. Magnetic resonance imaging (MRI) further revealed a significant reduction in the lesions associated with longitudinal extensive transverse myelitis. During the follow-up period, prednisolone was gradually tapered to a maintenance dose of 5-10 mg/day, whereas mycophenolate mofetil (MMF) was maintained at 1.0-1.5 g/day. The patient’s condition has remained stable for four years, with no signs of recurrence or progression observed on imaging examination. Therefore, this case suggests that protein A immunoadsorption may represent a potentially effective therapeutic option for patients with pSS-NMOSD who are refractory to conventional treatments.

## Introduction

Primary Sjögren’s syndrome (pSS) is a chronic autoimmune disorder characterized by inflammation and tissue destruction of the exocrine glands (1, 2). It can further manifest as various extraglandular complications, including neurological, pulmonary, and hematological involvement, etc. Amongst these complications, central nervous system (CNS) involvement is considered one of the most severe, significantly affecting patient prognosis and increasing mortality rate (3). Improving the therapeutic efficacy and outcomes of CNS involvement in pSS patients remains a challenging and poorly understood issue.

Neuromyelitis optica spectrum disorder (NMOSD) is an antibody-mediated autoimmune inflammatory demyelinating disease of the CNS, primarily affecting the optic nerves and spinal cord (4, 5). Thus far, research has shown that NMOSD may be related to AQP4-IgG seropositivity, is associated with various other types of autoimmune diseases, among which pSS is one of the most frequently reported systemic autoimmune diseases associated with NMOSD (6). Approximately 6.5% of NMOSD patients have been found to have comorbid Sjögren’s syndrome (7). Various approaches have been described for the treatment of primary Sjögren's syndrome (pSS) coexisting with neuromyelitis optica spectrum disorder (pSS-NMOSD), including high-dose glucocorticoid pulse therapy, plasma exchange, intravenous immunoglobulin injection (IVIG), and other immunomodulatory agents that are often employed to control inflammation and attenuate neurological symptoms (8). However, these therapies are limited, and are only partially effective in most cases (3). Herein, we present the case of a patient with pSS-NMOSD who benefited from protein A immunoadsorption combined with immunosuppressive treatment.

## Case presentation

A 35-year-old female who experienced mouth and eye dryness, right eye blindness, and decreased visual acuity in the left eye was initially diagnosed with pSS and optic neuritis at another hospital in October 2014. The principal abnormal test results were strongly positive for anti-ANA, anti-SSA, and anti-SSB antibodies, while rheumatoid factor (RF) were increased at 38 IU/mL. Levels of immunoglobulins (Ig)A (5.6 g/L), IgG (18.9 g/L), and IgM (2.6 g/L) were all elevated. Additionally, complement component (C)3 and C4 levels were decreased at 0.59 g/L and 0.082 g/L, respectively. The Schirmer test was positive. On admission, the patient had no light perception in her right eye, while visual acuity (VA) of the left eye was only 20/40. The VA of the left eye returned to 20/30 after a 3-day treatment with high-dose intravenous methylprednisolone (IVMP) (500 mg/d) and IVIG (0.4 g/kg/d), but her right eye only showed a little light perception. She was subsequently administered oral prednisone (starting dose 50 mg/d, with a gradual taper) and cyclophosphamide (CTX, 0.4g every two weeks) during the initial attack. Treatment was continued with the same regimen in the outpatient clinic. One month after discharge, the patient reported improvement in the symptoms of dry mouth and eyes. However, there was minimal change in the visual acuity (VA) of both eyes. Over the following six months, the patient’s condition remained stable, with the steroid dosage reduced to 10 mg/day. In September 2015, owing to significant menstrual irregularities, the CTX was adjusted to mycophenolate mofetil (MMF) 1.5g/d. Regrettably, the patient did not attend regular follow-up appointments and had not been taking medications consistently since September 2015.

**Figure 1:**
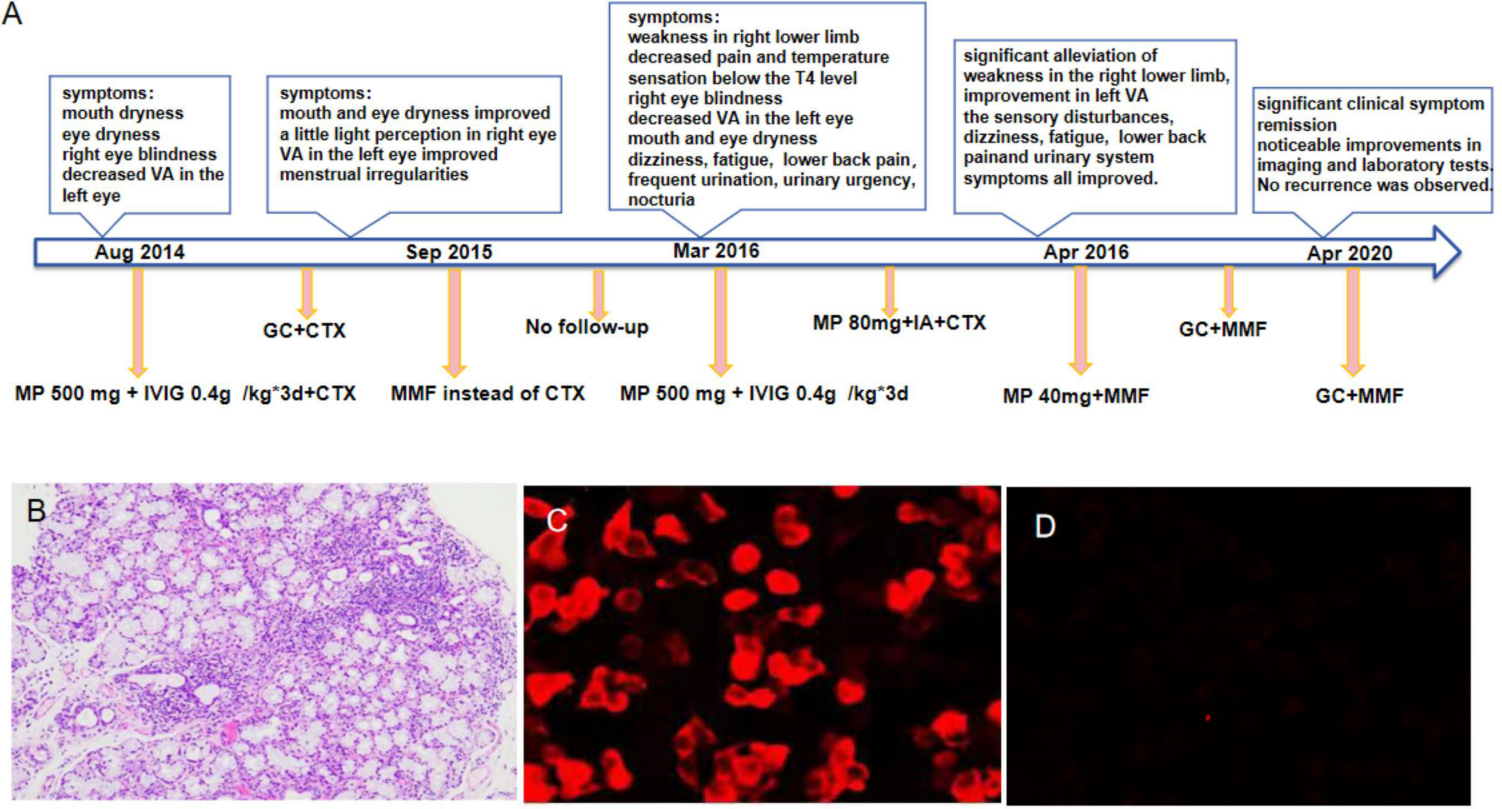
The treatment regimen for the patient, from the onset of the condition until the end of the follow-up period, is shown at different time points **(A)**. Histopathology of labial gland biopsy **(B)**. The changes in serum AQP4-IgG levels performed by cell-based assay before and after immunoadsorption treatment **(C, D)**. *GC, Glucocorticoid; CTX, Cyclophosphamide; MMF,Mycophenolate mofetil; VA,visual acuity. IVIG, intravenous immunoglobulin; MP, methylprednisolone;IA,immunoadsorption.

In March 2016, without any apparent trigger, the patient experienced a sudden onset of weakness in her right lower limb, accompanied by tingling and numbness. She also reported decreased visual acuity in the left eye, and blindness in the right eye. Other symptoms included mouth and eye dryness, dizziness, fatigue, frequent urination, urinary urgency, nocturia (5-6 times per night), and lower back pain. There was no indication of other autoimmune diseases in the patient’s personal or family history, and she denied any history of drug abuse or psychological disorders. The main positive findings on physical examination included the absence of light perception in the right eye and visual acuity of 2 m in the left eye. Muscle strength in both upper limbs was grade 5, that in the left lower limb was grade 5, and that in the right lower limb was grade 3. The pain and temperature sensation below the fourth left thoracic vertebra were also found to have decreased. Her EDSS score was six. **Figure 1A** shows the patient’ treatment regimen from the onset of the condition until the end of the follow-up period.

**Figure 2:**
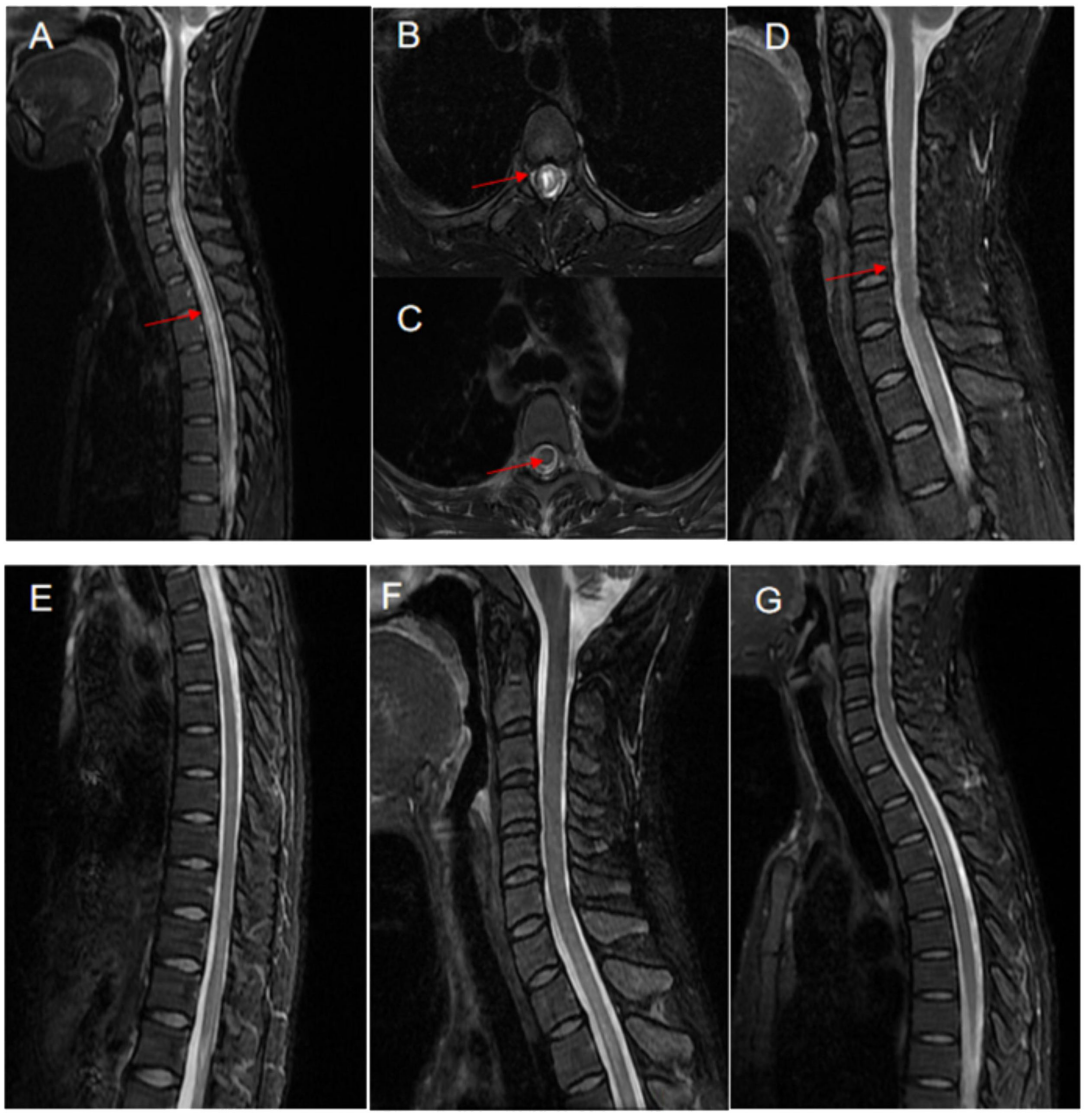
Neuroimaging changes of the patient with pSS-NMOSD patient. The MRI showed a T2 hyperintense lesion located at the cervical and thoracic spine levels of C5-T5 vertebrae **(A)** and also in the central portion of the spinal cord **(B)**, in March 2016 (indicated by arrows). After treatment with protein-A immunoadsorption combined with immunosuppressive therapy, there was a significant improvement in the previously noted signal abnormalities. as observed in the follow-up scans in April 2018 **(C–G)** and April 2020 **(F, G)**.

Auxiliary examinations: Salivary gland biopsy revealed two foci of lymphocyte invasion within the salivary gland tissue, with each focus containing more than 50 lymphocytes classified as grade IV. (**Figure 1B**). The Schirmer test was positive. The complete blood count (CBC) test indicated moderate anemia, with hemoglobin (Hb) levels at 80 g/L. Both the liver and renal function tests showed normal results. The erythrocyte sedimentation rate (ESR) was elevated at 147 mm/h. Rheumatoid factor (RF) was notably high at 418 IU/mL. Antibodies including anti-nuclear antibody (ANA), anti-SSA, anti-SSB, and anti-RO52 were all strongly positive, while anti-double-stranded DNA (anti-dsDNA), anti-Smith (anti-Sm) antibodies, and anti-cardiolipin antibodies were negative. Complement C3 and C4 levels were reduced at 0.69 g/L and 0.12 g/L, respectively. The levels of IgA, IgG and IgM were elevated, recorded at 8.15 g/L, 22.70 g/L, and 2.86 g/L, respectively. Additionally, cerebrospinal fluid (CSF) analysis indicated a normal cell count and normal levels of glucose, chloride, and oligoclonal bands. However, the protein level increased at 0.678 g/L. AQP4-IgG, as determined by the cell-based assay (CBA), was positive in the serum with a titer of 1:100 (**Figure 1C**), and turned negative after treatment (**Figure 1D**). The result in the cerebrospinal fluid (CSF) was negative. Furthermore, MRI of the cervical and thoracic spine showed a long, extensive T2-hyperintense lesion at the C5-T5 vertebral levels, involving the central portion of the spinal cord (**Figures 2A, B**). Ophthalmologic examination indicated that the right eye showed no reaction to the direct light reflex and only showed light perception. Normal light reflex was observed in the left eye. The fundus examination revealed optic atrophy of the right eye.

Through comprehensive clinical assessment, a diagnosis of pSS-NMOSD was established. She was initially treated with a high dose of IVMP (500 mg/day) and IVIGs (0.4 g) for 3 days, followed by immunotherapy with methylprednisone (80 mg for 1 d) and cyclophosphamide (0.4 g). However, her symptoms did not improve and, in fact, showed a tendency to worsen. There are no established treatment guidelines and specific recommendations for the treatment of pSS coexisting with NMOSD worldwide. After discussion with a neurologist, treatment with an increased dosage of methylprednisolone, plasmapheresis, immunoadsorption, or rituximab were considered. Unfortunately, due to patient’s significant side effects from corticosteroids, a plasma shortage and the high cost of rituximab, these plans were abandoned. In the meantime, we fortunately applied for the patient to receive complimentary access to an immunoadsorption column. After obtaining consent from the patient and her family, we decided to initiate combined treatment with immunoadsorption (IA). Methylprednisolone at 80 mg/day was continued throughout the IA treatment period. Although the original plan was to perform five sessions of IA, the patient experienced significant hypotension during the third session, which prevented her from completing the planned treatment. However, after the aggressive treatment mentioned above, the patient experienced gradual improvement in her left vision, significant alleviation of weakness in the right lower limb, and increased walking stability and strength compared to the pre-treatment level. The muscle strength on the left side gradually returned to grade 4+. In addition, the sensory disturbances on the left side and urinary system symptoms improved. Review of the patient’s laboratory indicators revealed an increase in hemoglobin level to 106 g/L, a decrease in RF to 45 IU/mL, and a reduction in ESR to 47 mm/h. The IgA and IgG decreased to 4.60 g/L and 15.20 g/L, respectively. Moreover, we observed a notable decrease in the levels of complement C3 and C4, which were reduced to 0.5 g/L and 0.09 g/L, respectively. The specific indicators are shown in **Supplementary Table S1**. Due to liver function re-evaluation showing an aspartate aminotransferase (AST) level of 89 IU/L and an alanine aminotransferase (ALT) level of 231 IU/L, therefore, we discontinued cyclophosphamide and switched to MMF 0.75 g twice daily as immunotherapy. Simultaneously, the dose of methylprednisolone was reduced from 80mg to 40 mg. Throughout the course of treatment, the patient’s muscle strength in the left lower limb improved to grade 5. In addition, we observed further improvements in pain and temperature sensation below the T4 level. Upon discharge, we re-examined the serum AQP4-IgG level, and found it to be negative (**Figure 1D**).

**Figure 3:**
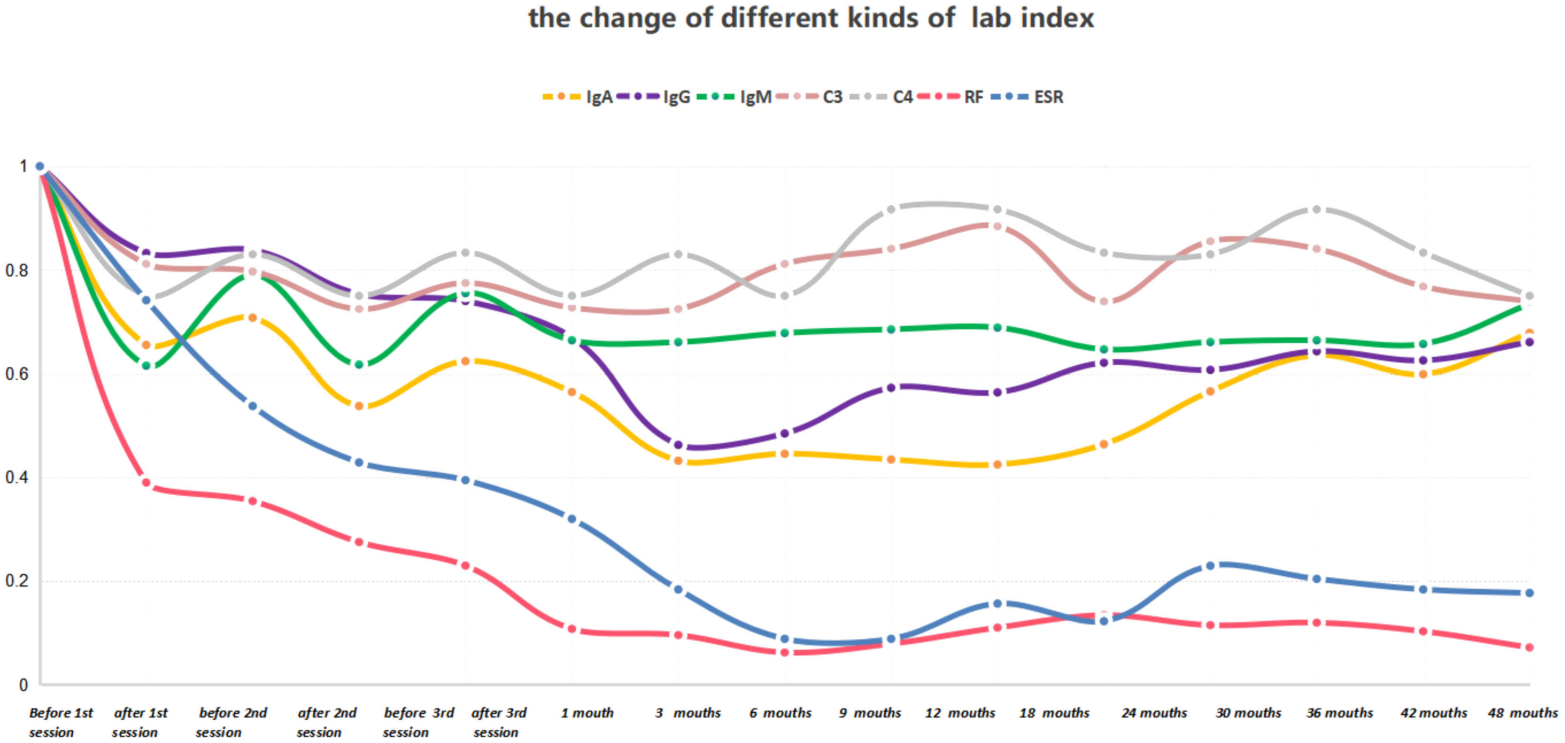
The serum concentrations of IgA, IgG, IgM, RF, ESR, C3, and C4 were measured before and after each treatment session, as well as at each follow-up time point. The laboratory indices before treatment were normalized to a value of 1.

After the patient was discharged, a comprehensive four-year follow-up was conducted. Throughout this period, the patient continued her treatment regimen consisting of prednisone and MMF, which effectively maintained her condition until the end of the monitoring period. In the best-case scenario, all indicators exhibited a satisfactory return to the normal range except for the consistently low levels of C3 and C4. Additionally, follow-up MRI scans conducted in April 2018 and April 2020 revealed a significant reduction in the intensity of high-signal areas of the spinal cord (**Figures 2C–G**). Simultaneously, we regularly monitored the serological markers associated with disease activity, including routine blood tests, RF, ESR, complement C3/C4, and immunoglobulin levels, as outlined in **Supplementary Table S1** and illustrated in **Figure 3**.

## Discussion

Neurological involvement in Sjögren’s syndrome (SS) encompasses a wide array of clinical manifestations, including hemiparesis, paraparesis, and blurred vision (3, 9). NMOSD is primarily characterized by severe optic neuritis (ON) and longitudinally extensive transverse myelitis (LETM), spanning multiple spinal cord segments. This condition predominantly affects young adults with a female predominance (10–12). NMOSD is often regarded as a disabling illness, with only a minor fraction of acute NMOSD episodes achieving full remission, while research has shown that approximately 70%-80% of patients are positive for AQP4-IgG expression (10–14). Over 80% of pSS patients who experience acute neurological events, such as myelitis or optic neuritis, have been reported to test positive for AQP4-IgG (14). Research has demonstrated that CNS impairment in conjunction with positive AQP4 antibody testing in pSS patients strongly indicates the coexistence of NMOSD. Furthermore, the presence of AQP4 antibodies is considered a high-risk factor for disease relapse in these individuals (15).

Here, we report the case of a patient who presented with xerostomia and xerophthalmia accompanied by positive ANA, anti-SSA, and anti-SSB titers in the blood. Additionally, salivary biopsy revealed a positive focus score and an abnormal Schirmer’s test, fulfilling the 2002 International Classification Criteria for Sjögren’s syndrome, as proposed by the American-European consensus group (16). Further, the patient simultaneously experienced optic neuritis with severe visual impairment in the right eye. During the disease repapse, involvement of the left eye and manifestations of acute myelitis, including weakness and paralysis of the right lower limb, paresthesia, and bladder dysfunction were also observed. Serum testing indicated positivity for AQP4-IgG, and in combination with the MRI findings, the diagnosis aligned with the criteria for NMOSD.

To date, the relationship between NMOSD and pSS has not been fully elucidated. Some clinicians have suggested that NMOSD may be a manifestation of CNS involvement stemming from pSS (17, 18). However, recent findings have revealed that in SS patients without NMOSD, there is no detectable expression of aquaporin-4 (AQP-4) antibody (19). This suggests that NMOSD is not directly involved in the CNS manifestations of pSS. Both pSS and NMOSD are antibody-mediated autoimmune diseases (8). Although the precise mechanism underlying the coexistence of pSS and NMOSD remains unclear, the formation of AQP4 antibodies is widely recognized as a crucial pathogenic factor in disease progression and clinical onset (20–22). Some researchers have speculated that anti-SSA antibodies and certain inflammatory factors in patients with pSS disrupt the blood-brain barrier via a mechanism involving vasculitis (8, 20). This disruption makes it easier for circulating AQP4 antibodies to target neural structures enriched with AQP-4, leading to NMOSD symptoms (23). Therefore, elimination of pathogenic antibodies is considered important for the treatment of patients with pSS-NMOSD.

Patients with PSS-NMOSD are typically treated with pulsed high-dose IVMP as the first-line therapy for the acute phase. Other immunosuppressive drugs, such as cyclophosphamide rituximab,mycophenolate mofetil, methotrexate, cyclosporine, and azathioprine are often used for long-term control of pSS-NMOSD (8, 24). Tocilizumab, an IL-6 receptor antagonist, is also considered a therapeutic option for patients with SS-NMOSD who display a poor response to rituximab (25, 26). Most patients respond well to glucocorticoid and immunosuppressive treatment; however, even with aggressive treatment, 90% of patients experience relapse within the first 3 years of the initial onset, while half of relapses occur within the first year (27). Studies have previously suggested that immunoadsorption (IA) may serve as a salvage treatment for NMOSD, particularly when high-dose IVMP therapy is ineffective. The 2019 revised American guidelines for blood purification recommend IA for the acute phase or relapse treatment of NMOSD (28). In **Table 1**, we provide a summary of the previous literature and the characteristics of reported cases of NMOSD or SS treated with IA therapy (13, 29–35). In one retrospective multicenter study, Kleiter et al. demonstrated that the clinical response rate in patients with NMOSD receiving IA therapy as an early intervention or subsequent treatment after other therapies achieved a remarkable 100% response rate (24). Subsequently, Faissner et al. conducted a retrospective study of IA as a monotherapy to treat seven cases of NMOSD attacks; they found that all patients’ symptoms improved with treatment without any instances of worsening (35). Moreover, in one case report, one patient with NMOSD was reported to have experienced a rapid reduction in circulating AQP4-IgG levels, immunoglobulins, and pro-inflammatory cytokines, coupled with an increase in lymphocyte counts (both T and B cells), following IA therapy (13). Although there have been few reports on the use of IA therapy for SS, Bohm et al. reported its use in treating severe SS as early as 2004 (29). Claus et al. reported the case of a patient with SS and recurrent pregnancy with a high risk of congenital heart block (CHB) in the fetus due to maternal anti-SSA/SSB antibodies. The patient ultimately underwent preventive IA treatment starting at week 19 of pregnancy, and delivered a healthy boy (30).

**Table 1:** Immunoadsorption therapy characteristics of SS or NMOSD patients.

References	Number of patients	Sex	Age	NMOSD or SS	Clinical Presentation	Positive of antibody	Treatment before IA	The therapeutic process of IA	Other drugs	outcomes	Side effect of IA
Böhm et al. (29)	1	F	38	pSS	Severe arthralgias and sicca symptoms	ANA、SSA、SSB、RF	Prednisolone(20mg/d) and methotrexate (25 mg a week)	Five sessions of IA therapy within a period of four weeks.	NA	Remarkable clinical improvement and remained stable at the 16th month follow-up.	NA
Claus et al. (30)	1	F	44	SS	One stillbirth and two spontaneous abortions	ANA、SSA、SSB	NA	36 sessions of IA therapy within a period of 18 weeks.	NA	Delivered a healthy boy	NA
Chen et al. (13)	1	F	29	NMOSD	Paraplegia and bilateral blindness without light perception	AQP4-IgG	IVMP(1000mg/d),3d	Five sessions of IA	MMF	Her symptoms did not improve at the end of the 5th session, but paraplegia and visual disturbance further ameliorated in the next 6-month follow-up.	NO
Nishimura et al. (31)	1	M	36	NMOSD	Reduction of visual acuity (VA)	AQP4-IgG	IVMP(1000mg/d),3d	Six sessions of IA	AZA	After the first course of treatment, the patient's vision improved, and AQP4-IgG turned negative. However, the patient was admitted again due to eye pain and vision deterioration, and was discharged after seven months. Following the second course of treatment, the eye pain improved, but there was no improvement in vision.	NO
Heigl et al. (32)	1	F	44	NMOSD	Unilateral optic neuritis,TM with sensory and mild motor deficits, bladder dysfunction.	AQP4-IgG	two high-dose steroid treatments (5×1000 mg, followed by 5× 2000 mg)	Six sessions of IA within two weeks, regular IA once every two weeks in two years	MTX, IVIG, RTX	The patient was clinically stable without disease progression or attacks. VA of the right eye was improved	headache, nausea,transient hypotension
Liu et al. (33)	1	F	36	NMOSD	Sudden loss of vision accompanied by postocular pain in the left eye.	AQP4-IgG	IVMP(500mg/d),3d,then MP 60mg	five sessions of IA	AZA	Light sensation was immediately recovered in the left eye following the first immunoadsorption (IA) treatment, and visual acuity (VA) in the right eye improved after the fifth IA treatment	NO
Liu et al. (34)	7	7F	27 to 64	NMOSD	Limb weakness and numbness were the main manifestations in four patients, and visual dysfunction was the main symptom in the other three patients.	AQP4-IgG	1 week of high-dose intravenous steroid therapy	five sessions of IA	MMF, and(or) RTX	The patient showed significant clinical improvement, imaging improvement, and a reduction in the AQP4-IgG levels.	transient hypotension
Faissner et al. (35)	10	6F/4M	22 to 49	NMOSD	Spastic tetraparesis was observed in four patients, unsteady gait in two patients, and visual dysfunction was the main symptom in five patients.	AQP4-IgG(5/10)	rituximab, tocilizumab, mitoxantrone	5.2 (range 3–7) cycles of IA	RTX, tocilizumab fingolimod, mitoxantrone	In the case of the seven patients, IA treatment resulted in improved VA for four, while the remaining three maintained stable VA.	NO

MMF, Mycophenolate mofetil; AZA, Azathioprine; CTX, Cyclophosphamide; RTX, rituximab; AQP4-IgG, aquaporin 4 antibody; VA, visual acuity; MP, methylprednisolone; NMOSD, Neuromyelitis optica spectrum disorder; TM, transverse myelitis; IVMP, high-dose intravenous methylprednison; IVIG, intravenous immunoglobulin.

In the present case, MRI revealed a long T2 signal at the C5-T5 vertebral levels and the presence of a positive AQP4 antibody, which suggests a severe and potentially recurrent condition. This implies a high risk of future paralysis and a poor prognosis. Optimal and effective treatment during the acute phase of NMOSD is crucial for long-term benefits, as failure to fully recover from acute attacks can exacerbate disabilities. IA therapy can improve symptoms of acute spinal cord injury and vision impairment, and in some cases, can even attenuate neurological symptoms after reaching a stable phase (32). Consistent with past research, our patient demonstrated significant clinical symptom remission following IA therapy, with noticeable improvements in imaging and laboratory tests. No recurrence was observed over a 4-year follow-up period.

Immunoadsorption therapy functions primarily by targeting and reducing the levels of self-antibodies, promoting the redistribution of antibodies, and modulating immune function. Relevant studies have shown that immunoadsorption can effectively decrease the concentration of AQP4-IgG antibodies in the blood, enabling a subsequent improvement in patients’clinical symptoms (13). In our patient, the AQP4-IgG antibody titer decreased from 1:100 to normal levels. We further observed significant reductions in complement C3 and C4 levels, RF levels, ESR, and immunoglobulin levels after IA treatment. The complement system plays an important role in the inflammatory processes of diseases, while it has been hypothesized that AQP4-IgG could trigger the complement cascade and cause complement-dependent cytotoxicity, leading to disruption of the blood-brain barrier, enhancing its permeability and exerting pathogenicity (13, 36). Complement depletion therapy may mitigate CNS damage by reducing the formation of the membrane attack complex. After the clearance of AQP4-IgG and other antibodies, the activation of the complement system may also be suppressed (13, 36). The clearance effect of IA on relevant pathogenic factors further confirms its effectiveness against NMOSD.

Nevertheless, adverse reactions may occur during immunoadsorption therapy. The most important adverse reactions reported by Koziolek et al. included infection of the central venous catheter, jugular vein thrombosis, chest pain, dyspnea, hypotension, rash, and decreased fibrinogen (35). In the present case, significant hypotension occurred during the third round of IA therapy, but was relieved following clinical intervention. Relevant studies have indicated that hypotension is associated with bradykinin and disease-induced autonomic dysfunction. Although adverse reactions can occur during IA therapy, serious consequences are rare, and most patients tolerate them well.

## Conclusions

Patients with concurrent SS and NMOSD experience more severe symptoms and poorer prognosis. Herein, we report for the first time that IA can rapidly and specifically eliminate pathogenic factors in patients with SS-NMOSD, significantly improving their clinical symptoms and effectively preserving the function of the affected organs. As such, IA could be considered a safe and effective treatment option. This case report highlights the potential advantages of IA for SS coexisting with NMOSD, particularly in patients who do not respond to conventional treatments. However, further clinical trials are required to provide additional data to assess the value of IA in patients with SS and NMOSD.

## Data availability statement

The raw data supporting the conclusions of this article will be made available by the authors, without undue reservation.

## Ethics statement

The studies involving humans were approved by the Second Affiliated Hospital of Xiamen Medical College. The studies were conducted in accordance with the local legislation and institutional requirements. The participants provided their written informed consent to participate in this study. Written informed consent was obtained from the individual(s) for the publication of any potentially identifiable images or data included in this article.

## Author contributions

WF: Data curation, Funding acquisition, Project administration, Writing – original draft, Writing – review & editing. XC: Data curation, Supervision, Writing – original draft, Writing – review & editing. PX: Formal analysis, Investigation, Methodology, Writing – original draft. BW: Conceptualization, Writing – original draft. YZ: Data curation, Formal analysis, Writing – original draft. JH: Data curation, Formal analysis, Writing – original draft. SW: Data curation, Writing – original draft. LL: Supervision, Writing – review & editing, Writing – original draft.
