# Supplementary material for: Protein-A immunoadsorption combined with immunosuppressive treatment in refractory primary Sjögren’s syndrome coexisting with NMOSD: a case report and literature review
Source: Front Immunol. 2024 Jul 11;15:1429405. doi: 10.3389/fimmu.2024.1429405 (PMC11269126; doi:10.3389/fimmu.2024.1429405)
Supplement: Supplementary file 1 [file Table_1.pdf]

TABLE 1 | The changes in serum concentration of IgA, IgG, IgM, RF,ESR,C3 and C4 levels before and after each session,as well as at follow-up time point.

Time point	IgA (g/L)	IgM (g/L)	IgG (g/L)	C3(g/L)	C4(g/L)	RF(IU/mL)	ESR（mm/hr）
before 1st session	8.15	22.7	2.86	0.69	0.12	418	147
after 1st session	5.34	18.9	1.76	0.56	0.09	163	109
before 2nd session	6.42	21.3	2.26	0.58	0.1	148	79
after 2nd session	4.38	17.1	1.48	0.5	0.09	115	63
before 3rd session	5.9	16.8	2.16	0.55	0.1	96	58
after 3rd session	4.6	15.2	1.9	0.5	0.09	45	47
1 mouth	3.52	10.5	1.89	0.5	0.1	40	27
3 mouths	3.63	11	1.94	0.56	0.09	26	13
6 mouths	3.54	13	1.96	0.58	0.11	33	13
9 mouths	3.95	12.8	1.97	0.61	0.11	46	23
12 mouths	3.78	14.1	1.85	0.51	0.1	56	18
18 mouths	4.61	13.8	1.89	0.59	0.10	48	33
24 mouths	5.18	14.6	1.9	0.58	0.11	50	30
36 mouths	4.88	14.2	1.88	0.53	0.10	43	27
48 mouths	5.53	15	2.1	0.51	0.09	30	26
